# Notes on Health Resorts

**Published:** 1899-12-09

**Authors:** Robert Saundby


### The Institutional Workshop.

### NOTES ON HEALTH RESORTS.

### MARTIGNY, CONTREXEVILLE, AND VITTEL.

By Robert Saundby, M.D., LL.D., F.R.C.P.

(*Continued from page* 153.)

The older part of Contrexéville is built along the banks of the Vair, a little stream which ultimately joins the Meuse. The peasant houses, covered with weathered tiles, brightened occasionally by a new red roof, are mingled with hotels, villas, and shops of modern construction, but the chief part of New Contrexéville lies between the river and the railway. Here are the principal hotels, and, above all, here is the Etablissement. In the park there is a covered promenade containing shops and seats, flower beds, and shady walks, a tennis court, croquet ground, shooting galleries, and other amusements. A pigeon-shooting ground and an embryo golf links are just outside the village. Visitors who are following the treatment rise about six a.m. in order to drink three or four glasses of water at the rate of a glass every quarter of an hour. The glasses hold 350 grammes, so that about three go to a litre. Meanwhile, the patients move about listening to the music of the band.

Ten o'clock is the hour for *dejeuner*, which, for people who have been up since six, is not too early; but there is no difficulty in getting meals served apart at any desired time. After breakfast the thoughtful soul chooses a shady place in which to take his coffee, or even retires to his room to escape the great heat, which is trying in the middle of the day. But the afternoon is also the time for driving, bicycling, and walking. For those who do not wish to walk far the zig-zag ascent of the Bellevue provides shade, air, and exercise. Upon reaching the summit there is a charming view over a foreground of rich agricultural country backed by densely-wooded hills.

Those who are taking the water cannot go far away, as at four they have to be back at the well for the afternoon dose. The band plays from three to five in the park, and at five o'clock many enjoy a douche, which is given to perfection by the excellent bath attendants. I have never experienced anything more delightful than the Contrexéville douche at the end of a sultry afternoon. After the douche one changes comfortably for dinner, which is at six. At six, too, the mail comes in, and there is a rush for home letters and London newspapers.

The evenings are by no means dull, for although the doctors discourage sitting about in the open air there is a theatre in the casino where a company performs on five nights in the week in a style to do no discredit to the stage of any metropolis, the actors being artists from Paris and Brussels, who in this way enjoy change of air and scene. When there is no theatre there is a concert, the music being supplied by the band and by artists who are making a tour of the watering places. Besides these amusements there are the more exciting attractions of baccarat and petits chevaux, or it is possible to arrange a quiet rubber of whist. Early hours are encouraged by the doctors, and by half-past ten there are very few people about; at eleven all lights are put out and the casino is closed.

The hours after breakfast at Contrexéville are clearly the part of the day which is least provided with rational amusement. There are very few carriages to be had, and those are about as bad as they can be. A supply of good carriages, and, above all, a coach to go daily excursions in the afternoon would be great additions to the comfort of the place. At present visitors at Contrexéville are dependent in some degree upon one another for amusement, but so many English resort there every year that there is no lack of agreeable society.

The waters of Contrexéville are valued for their action in cases of uric and oxalic acid calculi, of gravel, of pyelitis, and of catarrh of the bladder, of gouty glycosuria, of mild diabetes in elderly persons, and of gall stones.

The quantity of water prescribed is from two to four litres (three to six pints) daily, and it is usual to experience after drinking this quantity a well marked action upon the bowels. Where this is the case there can be only modified diuresis, but this does occur; in fact, the effect of the water is to dilute all the secretions of the body, and to wash the mucous surfaces over which they pass. Such a process must be cleansing and purifying, removing the various causes which have kept up inflammation, and dissolving and dissipating the small secretions which form the nuclei of larger stones, while facilitating the expulsion of those calculi which are too large to be readily dissolved. It is, however, very certain that the waters exercise a solvent action even upon calculi of some size, for some were shown to me by patients which presented a worm-eaten appearance, and were evidently undergoing disintegration. I was particularly impressed by the assurance I received from patients suffering from oxaluria that their condition had been really benefited by these waters, as every physician recognises the difficulty presented by these cases.

